# Global analysis of transcriptional regulators in *Staphylococcus aureus*

**DOI:** 10.1186/1471-2164-14-126

**Published:** 2013-02-26

**Authors:** Jose Antonio Ibarra, Ernesto Pérez-Rueda, Ronan K Carroll, Lindsey N Shaw

**Affiliations:** 1 Department of Cell Biology, Microbiology and Molecular Biology, University of South Florida, 4202 East Fowler Avenue, ISA 2015, Tampa, FL, 33620-5150, USA; 2 Departamento de Ingeniería Celular y Biocatálisis, Instituto de Biotecnologia UNAM, Av Universidad 2001, Cuernavaca, Morelos, CP 62210, Mexico; 3 Present address: Departamento de Microbiología, Escuela Nacional de Ciencias Biológicas, Instituto Politécnico Nacional, Prol. De Carpio y Plan de Ayala. Col. Sto. Tomás, México, DF, CP 11340, Mexico

**Keywords:** Transcriptional regulators, Virulence, Gene evolution, *Staphylococacceae*, Firmicutes

## Abstract

**Background:**

*Staphylococcus aureus* is a widely distributed human pathogen capable of infecting almost every ecological niche of the host. As a result, it is responsible for causing many different diseases. *S. aureus* has a vast array of virulence determinants whose expression is modulated by an intricate regulatory network, where transcriptional factors (TFs) are the primary elements. In this work, using diverse sequence analysis, we evaluated the repertoire of TFs and sigma factors in the community-associated methicillin resistant *S. aureus* (CA-MRSA) strain USA300-FPR3757.

**Results:**

A total of 135 TFs and sigma factors were identified and classified into 36 regulatory families. From these around 43% have been experimentally characterized to date, which demonstrates the significant work still at hand to unravel the regulatory network in place for this important pathogen. A comparison of the TF repertoire of *S. aureus* against 1209 sequenced bacterial genomes was carried out allowing us to identify a core set of orthologous TFs for the *Staphylococacceae*, and also allowing us to assign potential functions to previously uncharacterized TFs. Finally, the USA300 TFs were compared to those in eleven other *S. aureus* strains including: Newman, COL, JH1, JH9, MW2, Mu3, Mu50, N315, RF122, MRSA252 and MSSA476. We identify conserved TFs among these strains and suggest possible regulatory interactions.

**Conclusions:**

The analysis presented herein highlights the complexity of regulatory networks in *S. aureus* strains, identifies key conserved TFs among the *Staphylococacceae*, and offers unique insights into several as yet uncharacterized TFs.

## Background

*Staphylococcus aureus* is a facultative human pathogen and the casual agent of a diverse array of diseases, including superficial skin and wound-related tissue infections, food poisoning, bacteremia, endocarditis and pneumonia. This organism produces a diverse array of virulence factors, including toxins, adhesins, colonization and biofilm factors. *S. aureus* has obtained notoriety in recent years due to the appearance and worldwide spread of antibiotic resistant strains. Hospital associated (HA) and community associated (CA) infections caused by methicillin-resistant *S. aureus* (MRSA) have become a major public health concern, particularly for CA-MRSA infections as they cause life threatening disease in otherwise healthy individuals with no pre-existing risk factors [1]. Furthermore, CA-MRSA strains are replacing HA-MRSA strains in clinical settings, increasing the risk of transmission not only to patients but also into healthy individuals in the community (reviewed in [2]). As virulence determinant production is very tightly regulated in *S. aureus*, a thorough understanding of its regulatory network is necessary to fully comprehend the pathogenic processes of this bacterium. Additionally, exploring the regulatory differences between CA-MRSA and other MRSA strains may aid our understanding of the increase in virulence observed amongst community-associated isolates.

The relatively small size of Staphylococcal genomes, and their adaptability, suggests that these bacteria have a high degree of genome plasticity, depending on their environment [3,4]. Given the high number of virulence factors present in these bacteria, and the niche-specific role many of them play during different stages of the infectious process, gene expression must be finely tuned in order to efficiently coordinate their expression, and also continue to preserve energy pools. In this context, DNA-binding transcription factors (TFs) play an important regulatory role by either repressing or activating genes in response to environmental and physiological conditions.

Even though diverse strains of *S. aureus* have been extensively studied, and subjected to genome sequencing, the function of a large proportion of their genes remains unidentified. In this work, we define the TF repertoire for the CA-MRSA strain USA300-FPR3757 and classify it into regulatory families. We have evaluated the orthologous distribution of these elements in other sequenced bacterial genomes using the repertoire of TFs identified in USA300, and identified a core set of regulators for both the Firmicutes phylum, and the *Staphylococacceae* group. Finally, we examine the conservation of 135 USA300 TFs amongst 11 other *S. aureus* strains, identifying a key group of regulators that display a high degree of conservation, including many that have previously been demonstrated to play a role in virulence gene regulation. We also highlight cases whereby TFs are absent, or altered within strains, suggesting changes in the wiring of regulatory networks in individual isolates.

## Results

### Identification of TFs and σ factors in *S. aureus* USA300

**Figure 1:**
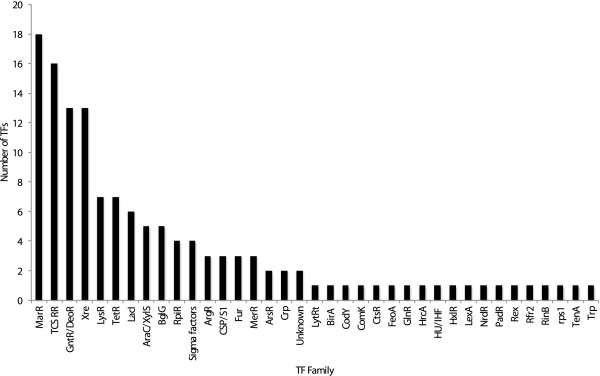
**Classification of transcriptional factors (TFs) in *S. aureus *strain USA300-FPR3757.** TFs were grouped after performing BLAST analyses and manual searches for several regulatory families. Those with no known family were placed in the “unknown” group.

**Figure 2:**
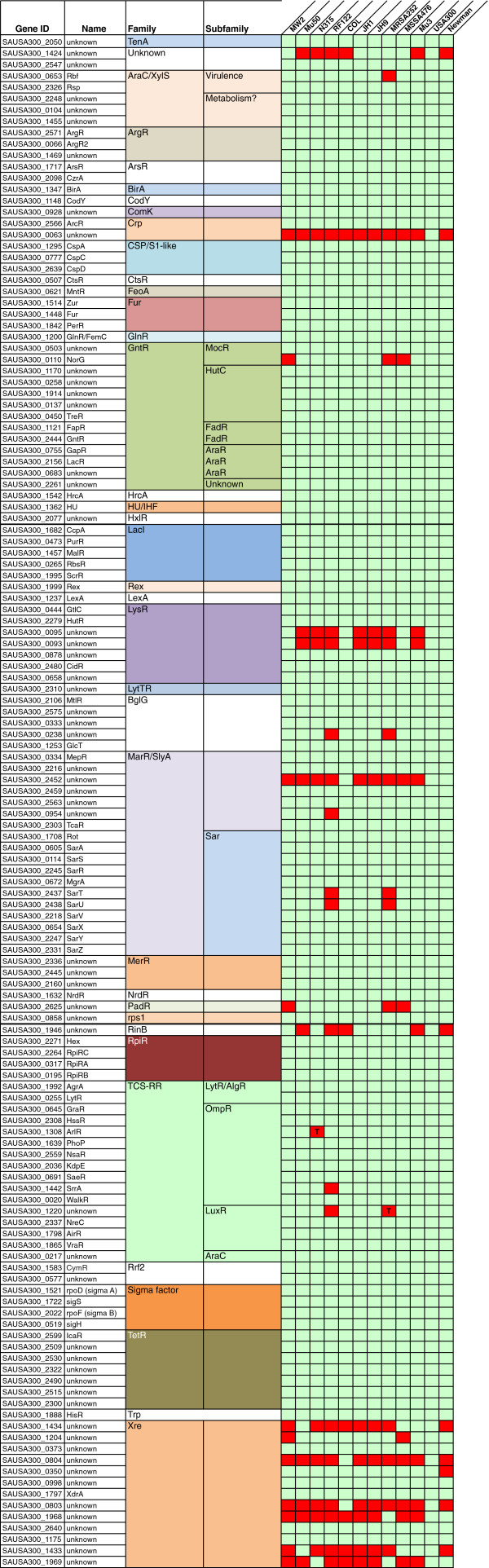
**Comparison of USA300-FPR transcriptional factors (TFs) with other *S. aureus *strains.** TFs identified in *S. aureus* USA300-FPR3757 were used to identify their orthologues in other *S. aureus* strains using BLAST and other tools (see Methods section). Presence of TFs is denoted by green and the absence by red colors; T indicates a truncated protein.

In order to identify the repertoire of TFs in *S. aureus* we focused on the recently emerged CA-MRSA strain USA300-FPR3757. This strain was selected for a number of reasons: Firstly, USA300 is the most prevalent CA-MRSA strain, associated with outbreaks in the USA, Canada and Europe [5,6]. Secondly, USA300 strains exhibit fewer genomic changes amongst isolated strains than other MRSA lineages, suggesting they originate from a common clone [7]. Finally, USA300 strains display hyper-virulence using various animal models of infection [8]. Therefore, we performed an extensive search for possible TFs using database assignments, Hidden Markov model (HMMs) profiles, BLAST similarities and literature searches. From this, 135 TFs and σ factors were identified in this strain. These putative regulators can be classified into 36 regulatory families (Figure 1 and 2), with only 2 classified as unknown. The largest TF family identified correspond to the MarR family (18 members), which includes the Sar subfamily; followed by the two component system response regulators (TCS-RR), with 16 members; followed by the GntR/DeoR family, and the Xre family (13 members each). Of interest, almost half of these elements have not been functionally characterized whatsoever (58 out of 135, 42.9%). For this reason, and to begin to understand the role of these uncharacterized TFs in *S. aureus*, we sought to explore potential functions for them by identifying similarities in sequence and genomic context with well-known proteins identified in other bacterial species. A complete summary of these findings is presented in Table 1. Collectively, 34 of the 58 uncharacterized TFs could be assigned a putative function via bioinformatics analysis. Together these results show the apparent variety of TFs in *S. aureus* USA300, and indicate that a large majority of them are not well-characterized. This presents an obvious gap, and the need for additional research to explore the complex, diverse and understudied regulatory circuits of this important human pathogen.

**Table 1:** **Possible role for uncharacterized TFs in *S. aureus *USA300**

**Protein ID**	**TF Family**	**Identities and comments**	**References**
SAUSA300_0063	Crp	Present in the ACME element	[9]
SAUSA300_0093	LysR	YwqM (30.2%) and GltR (27.9%), in *B. subtilis*. The latter appears to be involved in glutamate synthase expression.	[10]
SAUSA300_0095	LysR	PtxR of *Pseudomonas aeruginosa* PAO1 (41.5%) and *Yersinia pestis* (38.7%). Activates the expression of exotoxins and represses the expression of quorum sensing related genes.	[11,12]
SAUSA300_0104	AraC	Btr (24.5%) from *B. subtilis*. One-component regulator that controls siderophore transport	[13]
SAUSA300_0137	GntR	TreR (37%), involved in the regulation of trehalose related genes in *B. subtilis.* It is encoded divergent to purine synthesis genes.	[14]
SAUSA300_0217	TCS-RR	YesN (38.4%) and DegU (35.8%) from *B. subtilis*. The latter is involved in the expression of proteases and biofilm.	[15]
SAUSA300_0238	BglG	MnaR (25%) from *B. subtilis*	
SAUSA300_0258	GntR	LutR (44.4%), involved in regulation of lactate and biofilm in *B. subtilis.* It has a UbiC transcription regulator-associated (UTRA) domain.	[16]
SAUSA300_0333	BglG	LicR (28.5%) from *B. subtilis.* Regulates the transport and degradation of oligomeric beta-glucosides	[17]
SAUSA300_0350	Xre	YgzD (46%) *B. subtilis*	
SAUSA300_0373	Xre	No identity to characterized proteins	
SAUSA300_0503	GntR	YdeL (36%) and GabR (32.3%) both from *B. subtilis.* GabR regulates the expression of GABA synthesis genes. It also has some identity to *S. aureus* NorG (23%). It has a pyridoxal phosphate (PLP)-dependent aspartate aminotransferase domain.	[18]
SAUSA300_0577	GntR	This is the first gene in a putative operon with a pyridine nucleotide-disulphide oxidoreductase.	
SAUSA300_0658	LysR	*E. coli* OxyR (29.4%), positive regulator for a hydrogen peroxide-inducible regulon. Possible CcpC homolog, involved in regulation of TCA.	[19]
SAUSA300_0683	GntR	IolR (30.9%), repressor or the myo-inositol operon *in B. subtilis.* Its genomic context shows that it may regulate genes involved in fructose metabolism.	[20]
SAUSA300_0803	Xre	Toxin-antitoxin systems. These systems may contribute to the preservation of plasmids and genetic islands, however the role of many of them is still unknown	[21]
SAUSA300_0804	Xre	Toxin-antitoxin system	[21]
SAUSA300_0878	LysR	CytR (25%), regulator of the citrate synthase genes in *B. subtilis.* In *S. aureus* it is divergent to isopropylmalate synthase involved in Leu and pyruvate metabolism.	[22]
SAUSA300_0858	Rps1	*B. subtilis* YabR (42%), putative polyribonucleotide nucleotidyl transferase	
SAUSA300_0928	ComK	*B. subtilis* ComK (33.9%), required for genetic competence	[23]
SAUSA300_0954	MarR	YdgJ (35.4%), *B. subtilis*	
SAUSA300_0998	Xre	Rpc (33.7%) from *B. subtilis* bacteriophage phi 105. Involved in the regulation of lysogeny.	
SAUSA300_1170	GntR	YmfC (34.3%), *B. subtilis.* It has a UbiC transcription regulator-associated (UTRA) domain.	
SAUSA300_1174	GntR	YmfK (65%), *B. subtilis*	
SAUSA300_1175	GntR	YmfM (31.25%) *B. subtilis*	
SAUSA300_1204	Xre	No identity to characterized proteins	
SAUSA300_1220	TCS-RR	LuxR-like protein with identity to DesR (43.2%), responsible for thermosensing and signal transduction at low temperatures in *B. subtilis.* Also has identity to YvfU (45%) from *B. subtilis*	[24]
SAUSA300_1424	Unknown	No identity to characterized proteins	
SAUSA300_1433	Xre	No identity to characterized proteins	
SAUSA300_1434	Xre	Toxin-antitoxin system	[21]
SAUSA300_1455	AraC	AarP (30.8%), involved in regulation of 2'-N-acetyltransferase in *Providencia stuartii.*	[25]
SAUSA300_1469	ArgR	28% identity with *S.aureus* ArgR. In operon with a DNA repair protein	
SAUSA300_1914	GltR	*B. subtilis* YtrA (39.45%), possible repressor of an operon for a putative ATP-binding cassette transport system involved in acetoin utilization. YtrA is an additional regulator of cell envelope stress responses in *B. subtilis*.	[26,27]
SAUSA300_1946	RinB	RinB (76%) from phage 11. Activates *int* gene expression	[28]
SAUSA300_1968	Xre	No identity to characterized proteins	
SAUSA300_1969	Xre	LexA (28%), SOS regulator in *E. coli*	
SAUSA300_2077	HxlR	*B. subtilis* YodB (38.46%), regulation of *yocJ* (*azoR1*) after exposure to thiol-reactive compounds. A similar gene in *B. subtilis* regulates formaldehyde detoxification via *hxlAB*. In *S. aureus* it is not close to these genes, even though they are present in the genome.	[29]
SAUSA300_2106	BglG	ManR_(23.6%), mannose utilization in *B. subtilis*	[30]
SAUSA300_2160	MerR	AdhR (38%) *B. subtilis.* Transcriptional regulator involved in the response to aldehyde stress.	[31]
SAUSA300_2216	MarR	YwoH (31.6%) from *B. subtilis*	
SAUSA300_2248	AraC	*E. coli* YijO (28.6%), might be involved in the regulation of genes encoding enzymes related to PTS systems	[32]
SAUSA300_2261	GntR	No identity to characterized proteins	
SAUSA300_2300	TetR	No identity to characterized proteins. Divergent to 2 multidrug transport proteins (*emrAB* homologs)	
SAUSA300_2310	LytTr	Bears a LytTR domain, which is an only recently characterized family.	
SAUSA300_2322	TetR	*B. subtilis* YxbF (42.4%). In *S. aureus* it is in an operon with a CorA Mg transporter	
SAUSA300_2336	MerR	CueR (42.8%), involved in copper induction in *B. subtilis.*	[33]
SAUSA300_2445	MerR	36% identical to BltR, *B. subtilis*, and MerR (31%), *S. aureus.* The former is involved in response to structurally dissimilar drugs, while the latter is on a plasmid specifying resistance for mercurial compounds.	[34,35]
SAUSA300_2547	Unknown	*B. subtilis* YuaC (55.4%)	
SAUSA300_2452	MarR	Similar to *B. subtilis* YvnA (35.8%), (29%) and AdcR from *Streptococcus pneumoniae*. AdcR is able to sense metals for the regulation of zinc uptake proteins related genes encoding cell-surface zinc-binding pneumococcal histidine triad proteins and AdcAII (laminin binding). Also has a 33% identity to SarZ	[36]
SAUSA300_2459	MarR	MhqR (41.5%) regulates multiple dioxygenases/glyoxalases and an azoreductase that confer resistance to 2-methylhydroquinone and catechol in *B. subtilis*	[37]
SAUSA300_2490	LysR	No identities to characterized proteins. Divergent to operon encoding *mmpL* (transporter) and Feo iron dependent transporters	
SAUSA300_2509	TetR	*B. subtilis* YxbF (31.6%).	
SAUSA300_2515	TetR	SlmA (26.2%) in *Vibrio parahaemolyticus*. SlmA proteins are involved in nucleoid occlusion systems in *E. coli*. In *S. aureus* it is in an operon with genes encoding an oxidoreductase, an amidohyrolase and a hydrolase.	[38]
SAUSA300_2530	TetR	No identity to characterized proteins.	
SAUSA300_2563	MarR	PetP, (33.06%), necessary for photosynthetic and respiratory growth in *Rhodobacter capsulatus*	[39]
SAUSA300_2575	BglG	No identity to characterized proteins.	
SAUSA300_2640	Xre	ImmR (46% identity), involved in mobilization of the genetic element ICEB1 in *B. subtilis*	[40,41]
SAUSA300_2625	PadR	PadR (37.5%), repressor of phenolic acid response genes in *B. subtilis*	[42]

Amino acid sequences of uncharacterized TFs (Figure 2) were analyzed by using BLAST comparisons against the NR and SwissProt databases. In the third column is shown the closest identified protein (s), and their functional roles in corresponding organisms.

### Distribution of USA300 TF homologs in eubacterial species

Many bacterial TFs involved in key cellular processes are essential to the cell and are highly conserved. We hypothesized that a subset of the 135 TFs identified in USA300 would be conserved across eubacterial organisms. To test this hypothesis we set out to identify which TFs shared an orthologous protein in other bacterial phyla. A total of 1209 bacterial genomes were studied, comprising strains from the following phyla: Acidobacteria, Actinobacteria, Aquificae, Bacteroidetes, Chlamydiae, Chlorobi, Chloroflexi, Chrysiogenetes, Cyanobacteria, Deferribacteres, Deinococcus-Thermus, Dictyoglomi, division WWE1, Elusimicrobia, Fibrobacteres, Firmicutes, Fusobacteria, Gammatimonadetes, Nitrospirae, Planctomycetes, Proteobacteria, Spirochaetes, Synergistetes, Tenericutes, Thermobaculum, Thermatogae and Verrucomicrobia.

**Figure 3:**
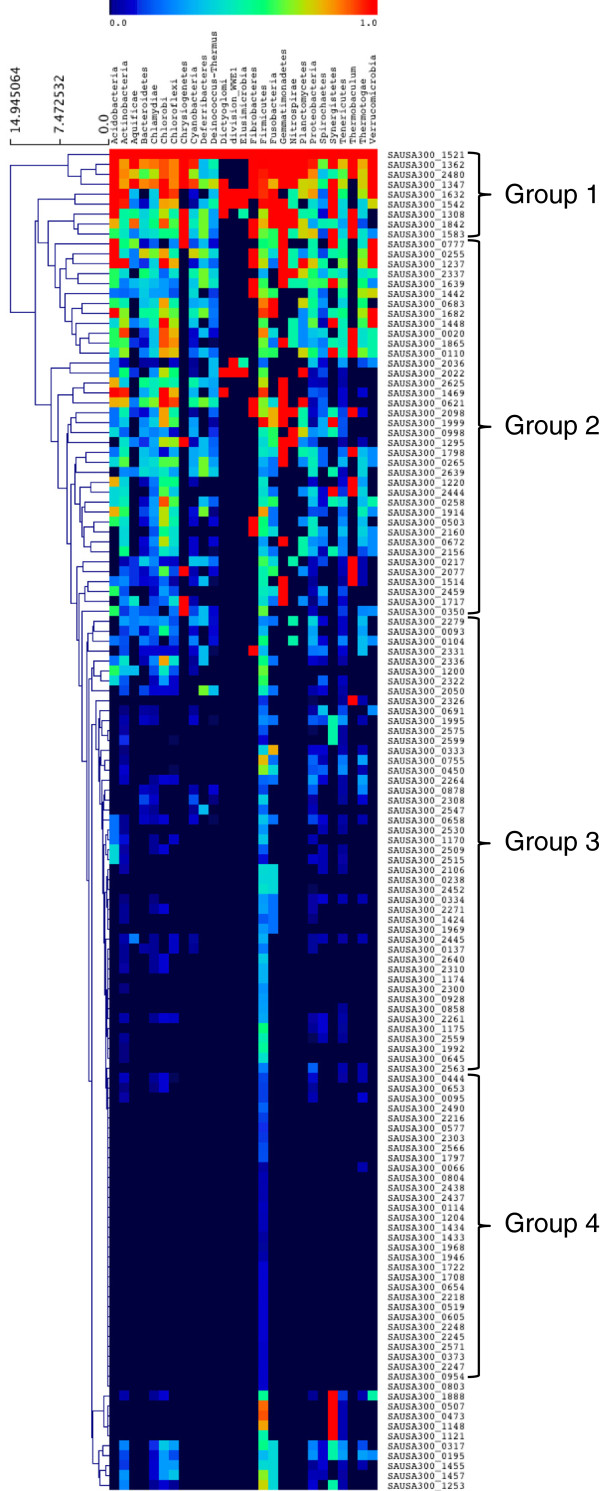
**Taxonomical distribution of *S. aureus *USA300 TFs in Bacteria.** The TFs found in *S. aureus* USA300-FPR3757 were used to identify their orthologues in the genomes of 1209 sequenced bacterial organisms. Results are presented as a heat map where 0 (purple) represents a low presence in any given domain and 1.0 (red) is a high presence. Shown are the accession numbers for TFs used for comparison (see also Figure 2, Table 1 and Additional file 1: Table S1 for more details).

Based on a clustering analysis we classified TFs into 4 main groups (Figure 3): Group 1 included orthologues highly conserved across most of the phyla (60-100% of organisms); Group 2, TFs less conserved in the diverse phyla analyzed (15–59%); Group 3 included mostly Firmicutes specific TFs (1–14%); and Group 4, those specific to *Staphylococacceae* (<1% of genomes, but abundant in this group). Nine proteins were found in group 1, suggesting an ancient origin for these regulators and perhaps playing a fundamental role in bacterial physiology (Additional file 1: Table S1). The one outstanding example of these is SAUSA300_1521 (sigma A, σ^A^), essential for housekeeping transcription in bacteria. Two proteins less conserved in this group are SAUSA300_1362 (HU) and SAUSA300_2480 (CidR), involved in genome packing, and the regulation of murein synthesis gene expression, respectively; although they are less conserved in Dictyoglomi, division WWE1 and Elusimicrobia. This suggests that organisms in those groups perhaps use alternative proteins to package their DNA. Group 2 includes several proteins widely distributed amongst all bacterial phyla, except in organisms with smaller genomes where gene loss appears to have occurred, such as in *Dictyoglomus turgidum* and *Bifidobacterium animalis* ssp. *animalis*, amongst others (reviewed in [43]). Examples of these proteins are SAUSA300_1632 (transcriptional repressor of ribonucleotide reductases genes, NrdR) and HrcA (SAUSA300_1542), a regulator of genes involved in heat-shock. The third group of proteins was identified as being conserved mainly in the Firmicutes phylum. This group includes NsaR, GraR, and AgrA, proteins that serve as the response regulator of two-component systems, GapR and TreR (GntR-like proteins involved in the regulation of metabolism related genes), GlnR (glutamine synthetase repressor), HutR (repressor of the histidine utilization operon, *hut*) and SarZ (which promotes the expression of virulence genes), as well as other as yet uncharacterized proteins. Group 4 includes TFs with very few homologues outside the *Staphylococacceae* family, and thus represents *Staphylococacceae* specific TFs. This group contains most of the members of the Sar family (Rot, SarA, SarV, SarY, SarR, SarU, SarT, SarS and SarX), the alternative sigma factor σ^S^, and as yet uncharacterized TFs from the MarR, AraC/XylS and Xre families.

As such, by using the TFs found in *S. aureus* USA300 as a scaffold to interrogate other sequenced bacterial genomes, we were able to identify: (i) TFs that are conserved in all the bacterial phyla, suggesting an ancient origin and critical cellular function, (ii) those regulators found mainly in the Firmicutes, and (iii) regulators found exclusively in the *Staphylococacceae*.

### Comparison of TFs between *S. aureus* strains

**Figure 4:**
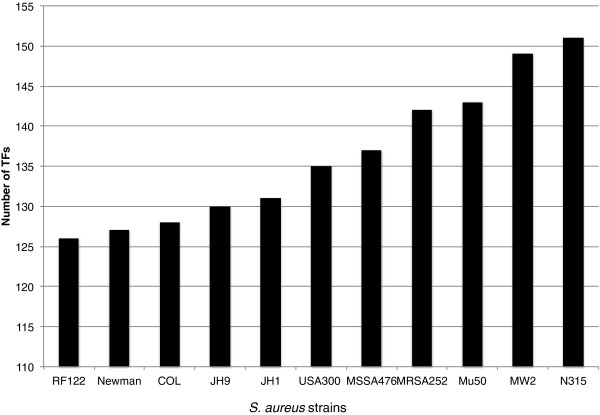
**Total number of TFs in multiple *S. aureus *strains.** TFs in all *S. aureus* strains were detected using a similar method to that used for strain USA300-FPR-3757.

To further explore TFs conserved in the *Staphylococacceae*, and identify those that are potentially involved in the regulation of virulence gene expression, we compared the USA300 TFs with those in other Staphylococcal strains. Eleven additional *S. aureus* strains were examined (Additional file 1: Table S2); ten of which are human specific pathogens, while one (RF122) is a pathogen of cattle, and has important agricultural implications. These strains were selected as they have been extensively studied, and are representative of the wide genetic variability across *S. aureus* strains. We determined that the total number of TFs and σ factors varies from strain to strain, ranging from 126 for the bovine pathogenic strain RF122, to 151 for the MRSA strain N315 (Figure 4). This variation among different strains is most likely the result of genomic rearrangements, duplications, and the acquisition of novel genetic elements, such as phages and pathogenicity islands [44].

A total of 112 TFs were identified as being present in all *S. aureus* strains, including regulators of genes involved in metabolic (ArcR, Fur, HutR, GntR, GlnR, CcpA, ArgR, ArcR, FeoA, Fur, PerR, FemC, TreR, GapR, LacR, FapR, CcpA, PurR, HisR, and multiple TCS) and virulence processes (AgrA, SarA, SarR, SarS, SarV, SarX, SarY, SarZ, Rot and MgrA). The high degree of conservation of these TFs probably emphasizes the need for specific and precise regulation of genes involved in these key physiological processes. In addition, we found TFs in this group that are associated with genome homeostasis such as LexA and HU that respond to DNA damage and structure, respectively. Unsurprisingly, given their role in transcription processes, all the σ factors (σ^A^, σ^B^, σ^H^ and σ^S^) were conserved across all strains.

Conversely, a number of TFs were found in most strains, but were absent in one or two. The absence of these TFs suggests that their loss leads to a difference in the strain specific regulation of important pathways. Amongst this group are the TCS-RRs. Fourteen of the sixteen *S. aureus* TCS are conserved in all strains analyzed, however ArlR, for example, is present, yet truncated, in strain N315. Similarly, the uncharacterized SAUSA300_1220 is truncated in strain MRSA252; and both SAUSA300_1220 and SrrA are absent from the bovine pathogen RF122 (Figure 2). Interestingly, for these latter two regulators, the sensor histidine kinase is also missing. Additionally, a rare event occurs where the TCS KdpDE is duplicated on SCC*mec* II; thus strains N315, Mu50, JH1, JH9, Mu3 and MRSA252 are unique in carrying two copies of this regulatory system. The occurrence of this duplication has previously been observed [45], however its biological significance is not yet clear.

In the context of non-TCS-RR, other TFs are also variable across *S. aureus* strains, including SAUSA300_0063, which is only found in USA300. This TF is a likely duplication of the ArgR arginine repressor, and is encoded on the arginine catabolic mobile element (ACME), which is present only in USA300 strains and is linked to SCC*mec* IV [46]. By far, the most variability within a family of TFs was observed for the Xre-like elements. This family includes regulators in Eukaryotes, Archaea and Bacteria, and is evolutionarily related to the bacteriophage regulators Cro and cI [47]. Our analysis showed that *S. aureus* USA300-FPR3757 has 13 putative members of this family. To our knowledge XdrA, which is present in all strains analyzed and serves as an activator of the virulence factor protein A [48], is the only member of this family that has been characterized. In contrast to some of the Xre-like proteins found herein, XrdA is not encoded on or near a phage-related element. Other Xre regulators also exist that are similarly unassociated with lysogenic bacteriophages, including SAUSA300_0804, SAUSA300_2640 and SAUSA300_0998. In total, five Xre-like TFs were found in all *S. aureus* strains, and appear to be unassociated with phage-like elements. It is tempting to suggest that the presence or absence of these Xre elements could be considered a genetic fingerprint for each of the strains, and may influence regulatory network in subtle yet wide-reaching ways.

## Discussion

The overall aim of this study was to gain insight into the composition and conservation of TFs in the *Staphylococacceae*, specifically in the major human pathogen, *S. aureus*. First we detected TFs in the USA300-FPR3757 strain, identifying 135 elements belonging to 36 different regulatory families. Of note, almost half of these (58 out of 135, or 42.9%) have yet to be characterized. Herein we were able to propose possible roles for most of them, leaving only 9 without ascribed or predicted functions.

The most abundant TFs in this strain belonged to the MarR family, which includes the Sar-like subfamily [49]. One such TF (SAUSA300_2452) was of particular interest as it showed 33% identity with SarZ over 64% of the length of the protein, as determined using BLASTP, suggesting that it might be a new member of this family. In order to corroborate whether this protein is related to the Sar family we generated a phylogenetic tree with all known Sar and MarR proteins found in the USA300 strain and compared them with the crystal structure of MgrA. As seen in Additional file 2: Figure S1, MarR-like TFs were grouped in three clades: one including SarX, TcaR and four non-characterized MarR proteins; a second included SarA, SarY, SarR, SarS, SarU, SarV and Rot; and the third included MgrA, SarZ and SAUSA300_2452. From this analysis it seems that SAUSA300_2452 is phylogenetically related to SarZ and MgrA, suggesting it may belong to this subfamily.

Given the adaptability of *S. aureus* to multiple environments it is perhaps no surprise to find that TCS-RR family was one of the most abundant families, with 16 members. Despite the fact that this group of proteins has been widely studied, two members remain uncharacterized, SAUSA300_1220 and SAUSA300_0217. SAUSA300_1220 shares homology with *B. subtilis* DesR (43% identity over 99% of the protein, as determined using BLASTP), which is involved in sensing changes in temperature and regulating the expression of genes that respond to this environmental cue [24]. Of note, in gamma-proteobacteria this role is accomplished by the histone-like protein H-NS and other related factors, which are seemingly absent in the firmicutes [50]. Indeed, in *S. aureus* there is only one protein related to the histone-like family, suggesting that regulation of the thermal response is achieved by other TFs, which may include SAUSA300_1220. SAUSA300_0217 has some identity to DegU (35.8% identity over 42% of the protein, as determined using BLASTP) from *B. subtilis*, which is involved in the modulation of protease expression and biofilm formation. Importantly, there is some suggestion that this system, or rather its counterpart in strain COL, is expressed during anaerobiosis [51].

We also used the USA300 TFs as a scaffold to define how conserved these regulators are within eubacterial species. This analysis showed that nine TFs have orthologues in almost all of the 1209 genomes analyzed. This suggests that these proteins have an important role in cell fitness, and have a common ancestral origin. Included in this group are: SAUSA300_1521, the primary sigma factor σ^70^ (σ^A^) that drives house-keeping gene expression; and the histone-like protein HU (SAUSA300_1362), which is important in controlling DNA structure [50]. Also included in this group is SAUSA300_1347 (BirA), which is involved in regulation and biotinylation of the essential metabolic factor CoA [52]. CidR (SAUSA300_2480) regulates the expression of holin/anti-holin complexes involved in peptidoglycan synthesis, and is therefore important for bacterial survival, at least in *S. aureus* strains [53]. SAUSA300_1632 is a NrdR orthologue that regulates the expression of ribonucleotide reductases, necessary for DNA and RNA synthesis [54]. HrcA (SAUSA300_1542) is an important regulator of proteins involved in the heat-shock response in *Bacillus subtilis*[55]. Though less conserved than the other TFs in group 1, ArlR (SAUSA300_1308) is still preserved in many bacterial phyla. ArlR is a two-component response regulator that controls the expression of 114 genes in *S. aureus*, including those involved in cell division and growth [56].

We also identified a group of regulatory proteins whose orthologues are conserved within most Firmicutes, and are involved in processes such as metabolism (GapR, TreR, GlnR, HutR, Hex, RpiRC, ScrR), stress response (NsaR, GraR, MepR) and virulence (AgrA, SarZ, SaeR, IcaR, Rsp). The prevalence of TFs related to metabolism and stress in so many Firmicutes would be expected as this suggests a common origin. It is interesting to note that while highly conserved regulators are involved in key cellular processes, TFs that are phylum specific are involved in more specialized functions i.e. stress response and virulence. For example, it is possible that in non-pathogenic organisms, those TFs known to regulate virulence genes in other species serve to control genes for niche adaptation or symbiosis.

At the most specific level, we defined those TFs that were conserved uniquely in the *Staphylococacceae*. Most of the TFs in this group are related to virulence and environment adaptation, including the Sar family of proteins, the alternative sigma factor SigS, and some elements involved in metabolism. Collectively, and to our knowledge, this is the first global study that circumscribes the TFs for the Firmicutes, and more specifically, the *Staphylococacceae*.

Another of our objectives was to define how conserved TFs are across multiple, well-characterized *S. aureus* strains. We first identified the TFs for eleven additional strains (Figure 4, and data not shown) and then compared them with those in USA300. The majority of the TFs were conserved across all strains (83%), which is largely comprised of those that are part of the core Staphylococcal TF suite. The absence of the other 17% of regulatory proteins indicates that these are not central for survival or pathogenesis, and may be responsible for subtle, strain specific, fine-tuning of gene expression patterns. For instance, SAUSA300_0063 is a Crp-like TF encoded in the ACME region. ACME has been found only in USA300 strains, and is thought to play a role in virulence [46,57]. All known USA300 strains have this genetic element, supporting its role in virulence processes and/or transmission. In contrast, Rbf, an AraC-like protein that positively regulates biofilm formation [58] is present in all strains except MRSA252. This exemplifies our contention that differences in regulatory networks adapt to strain specific process. For example, MRSA252 is a robust biofilm forming isolate, yet is still capable of undergoing this process in the absence of Rbf. This suggests that this process is multifactorial, involving many different regulators, and is adaptable within strains to individual growth or pathogenic environments.

The biggest difference in TFs amongst Staphylococcal strains was observed in phage related regulators. These demonstrated the most variability, which is unsurprising, as each strain has acquired variable phage content over time. Despite the fact that they are located on phage elements, some have developed a key role in the regulation of virulence genes in the core chromosome. Such is the case of XdrA, which regulates the expression of protein A, an important immune evasion virulence factor. Moreover, some TFs are located in the vicinity of putative toxin-antitoxin systems, e.g. SAUSA300_2640; such systems have been suggested to contribute to the preservation of plasmids and genetic islands [21]. Additionally, one of these TFs (SAUSA300_0998) is located close to a putative putrescine secretion system, possibly forming an operon, suggesting it might be involved in its regulation.

We also compared TF variation from human-specific *S. aureus* strains to the bovine adapted isolate RF122, which is associated with mastitis in cattle. Such strains are of significant environmental and economic importance as they are responsible for massive losses financially in animal production each year [59]. We hypothesized that the difference in host preference for RF122 would coincide with altered TF content when compared to human adapted strains of *S. aureus*. A comparison of the TFs from USA300 with those in RF122 identified 17 elements that were unique to human versus bovine strains (Table 2). While it is difficult to predict the combined outcome on gene expression this variation would have, certain examples suggest changes to virulence gene expression in RF122 that may account for altered species specificity. An example of this is the loss of SarT and SarU in RF122 (quoted in [60]). SarT influences the expression of *sarS*, and hence that of *spa*, while at the same time repressing the expression of α-toxin (*hla*), *sarU* and *agr*[61,62]. Hla is a central virulence factor, known to be important for infection in animal models of disease causation [63]. Thus, given the absence of SarT, it is possible that Hla is upregulated in RF122. In support of this, a recent study of multiple bovine *S. aureus* isolates, including RF122, revealed increased production of Hla [64]. Indeed, it was shown that overexpression of *hla* was not only due to the presence of SNPs, but also upregulation by SarZ, and possibly by the elevated expression of other regulators such as AgrA, SaeR and ArlR. An additional 9 TFs were identified in RF122 that were not present in USA300 (Additional file 1: Table S1). Many of these are Xre family proteins, and are associated with horizontally acquired DNA. Studies have previously shown that RF122 has a distinctive pathogenicity island (SaPIbov) [65], therefore it is possible that these horizontally acquired TFs, together with additional virulence genes in RF122, form a regulatory network that governs host specificity. As we have demonstrated here, although RF122 lacks some TFs, which in turn has the capacity to render it more virulent, it also has additional TFs that could potentially regulate well-known virulence genes, and maybe those in strain-specific genetic regions such as SaPIbov.

**Table 2:** TFs specific to USA300 and RF122

**TFs present only in RF122**	**USA300 TFs absent in RF122**
SAB2083c	SrrA
SAB1911	SAUSA300_1220
SAB1910	SAUSA300_1424
SAB1750c	SAUSA300_0093
SAB1757	SAUSA300_0954
SAB1836c	SAUSA300_0095
SAB1297	SAUSA300_0238
SAB1256c	SAUSA300_2452
	SAUSA300_0858
	SarT
	SarU
	SAUSA300_1434
	SAUSA300_0804
	SAUSA300_0803
	SAUSA300_1968
	SAUSA300_1433
	SAUSA300_1969

## Conclusions

In summary, the analysis presented herein demonstrates the incredible complexity of regulatory networks and gene regulation in *S. aureus*, and offers unique insights into many as yet uncharacterized TFs in this important human pathogen. A comparison of *S. aureus* TFs with those of other bacterial phyla reveals two main types of TF in Staphylococci. The first group represents a core of regulators, present in common ancestors of diverse bacteria that participates in the regulation of key cellular processes. The second group represents TFs whose function seems to be genus/species specific (e.g. virulence gene regulators and those for specific metabolic requirements). Therefore we propose that TFs in group 4 forms the core set of TFs in the *Staphylococcaceae*. Included in this group are most the Sar regulators, which are part of the MarR family, and other, as yet uncharacterized proteins. Additionally, we focused on the differences amongst well-characterized *S. aureus* strains and found absence of TFs that might dictate changes in regulatory networks for each isolate. Finally, the similarities and differences in TF content between the human pathogen USA300 and the bovine pathogen RF122 were determined. Previous reports have shown that the expression of virulence factors amongst bovine and human isolates is different, and here we observed differences in the TFs content for these two strains. It is possible that some of these elements are involved in differentially regulating virulence factors, perhaps through modulation of known elements such as AgrA and SarA.

## Methods

### Identification of DNA-binding transcription factors

The complete genomes of twelve *S. aureus* strains were obtained from ftp://ftp.ncbi.nlm.nih.gov/genomes/Bacteria. Open reading frames that encode predicted protein sequences*,* i.e. the proteome in all bacteria, were considered as annotated genes. In order to identify the repertoire of TFs in *S. aureus* strains, domain assignations associated to DNA-binding regions in the Superfamily database (25-Apr-2010 version), and others identified and annotated in PFAM [66] were used*.* Additionally, family-specific Hidden Markov Models (HMM) constructed from three bacterial models: *Escherichia coli* K-12, *Bacillus subtilis*, and *Corynebacterium glutamicum* were used to search *S. aureus* genomes. Briefly, 90 family-specific HMMs previously reported for *E. coli* K-12 [67] and 57 family-specific HMMs from *B. subtilis*[68] were used to scan complete genome sequences (E-value threshold of 10^-3^), with the “*hmmsearch*” module from the HMMer suite of programs (http://hmmer.janelia.org/). These HMMs were constructed using the previously identified TF families in *E. coli* K-12 and *B. subtilis* as seeds, considering the DNA-binding domain (DBD) sequence (around 60 amino acids) of every protein from multiple families. *S. aureus* USA300 proteome sequences were scanned with these HMMs, and proteins with less than 60% coverage in the DNA-binding region against their corresponding HMM were excluded. Finally, regulators deposited in the DBD database [69] were also considered as potential DNA-binding TFs.

In order to evaluate the distribution of TFs and their corresponding orthologues across all bacterial genomes, a hierarchical average linkage-clustering algorithm was applied with a Manhattan correlation distance as a similarity measure. Analyses were performed using the program Mev4 (multi-experiment viewer; PMID:12613259). In order to determine the relative abundance of TFs and their orthologues, we calculated the fraction of genomes in the group that had at least one member versus the number of representative organisms. Thus, the following formula was considered: relative abundance by phylum (total number of orthologues identified)/(total number of organisms by phylum). Thus, a value of 1 corresponds to presence and 0 represents absence. Because our aim was to evaluate the taxonomical distribution of orthologues proteins, 27 taxonomical phyla corresponding to eubacteria were considered.

In order to achieve comparative analysis strain USA300-FPR3757 was used for the classification of TFs into evolutionary families. This was based on PFAM annotations, and corroborated using BLAST searches (using default conditions) against well-annotated protein families.

### Comparison of ORFomes from different *S. aureus* strains

Based on the USA300-FPR3757 ORFome, we searched for the presence and absence of TFs in eleven different strains, including: Newman, COL, JH1, JH9, MW2, Mu50, Mu3, N315, RF122, MRSA252 and MSSA476. This comparison was achieved by sequence analysis using the Comprehensive Microbial Resources (CRM) database from JCVI (http://cmr.jcvi.org/tigr-scripts/CMR/CmrHomePage.cgi), and confirmed by BLAST searches.

Additionally, in order to evaluate the phylogenetic distribution, the *S. aureus* TF repertoire was used to identify orthologous proteins in 1209 sequenced eubacterial strains. Orthologous relationships were identified based on BLASTP reciprocal best hits, with an E-value cut-off of ≤ 1e-6, as described elsewhere [70]. Finally, the phylogenetic distribution of each TF was evaluated based on a hierarchical cluster analysis.

## Competing interests

The authors declare that they have no competing interests.

## Authors’ contributions

JAI and EP-R conceived the study and its coordination. EP-R performed all the bioinformatics analysis, including large-scale sequence analysis; JAI helped in bioinformatics analysis and data mining. RKC and LNS helped in the design of the study. JAI, EP-R, RKC and LNS drafted and wrote the manuscript. All authors read and approved the final manuscript.

## Supplementary Material

Additional file 1: Table S1 *S. aureus* USA300 TFs conserved in eubacteria. Groups are defined as: 1, proteins with orthologues in 60–100% of genomes studied; 2, 15-59%; 3, 1–14%; and 4 <1% of genomes. **Table S2.***S. aureus* strains analyzed in this work. Click here for file

Additional file 2: Figure S1 Phylogenetic tree for the MarR-like proteins. Sar and MarR proteins, along with uncharecterized MarR proteins, were aligned using the MgrA crystal (2BV6) structure as a template. Three clades are denoted, see text for details. SarU, SAUSA300_1114 and SAUSA300_2247 exhibit two MgrA-like domains and therefore are shown as duplicated in the tree. Click here for file
