# Supplementary material for: Global analysis of transcriptional regulators in Staphylococcus aureus
Source: BMC Genomics. 2013 Feb 26;14:126. doi: 10.1186/1471-2164-14-126 (PMC3616918; doi:10.1186/1471-2164-14-126)
Supplement: Additional file 1: Table S1 — S. aureus USA300 TFs conserved in eubacteria. Groups are defined as: 1, proteins with orthologues in 60–100% of genomes studied; 2, 15-59%; 3, 1–14%; and 4 <1% of genomes. Table S2.S. aureus strains analyzed in this work. [file 1471-2164-14-126-S1.doc]

**Supplementary Table 1. *S. aureus* USA300 TFs conserved in eubacteria.** Groups are defined as: 1, proteins with orthologous in 60-100 % of genomes studied; 2, 15-59%; 3, 1-14 %; and 4 <1% of genomes.

| **Group 1** | **Group 2** | **Group 3** | Group 4 |
| --- | --- | --- | --- |
| | SAUSA300_1521 | | --- | | SAUSA300_1362 | | SAUSA300_1347 | | SAUSA300_2480 | | SAUSA300_1632 | | SAUSA300_1308 | | SAUSA300_1842 | | SAUSA300_1542 | | SAUSA300_1583 | | | SAUSA300_0255 | | --- | | SAUSA300_1237 | | SAUSA300_1639 | | SAUSA300_0621 | | SAUSA300_1682 | | SAUSA300_0110 | | SAUSA300_2337 | | SAUSA300_0777 | | SAUSA300_2098 | | SAUSA300_1865 | | SAUSA300_1448 | | SAUSA300_0020 | | SAUSA300_1469 | | SAUSA300_0683 | | SAUSA300_0265 | | SAUSA300_1999 | | SAUSA300_2022 | | SAUSA300_1295 | | SAUSA300_1798 | | SAUSA300_1442 | | SAUSA300_0258 | | SAUSA300_2625 | | SAUSA300_1914 | | SAUSA300_0998 | | SAUSA300_2444 | | SAUSA300_0503 | | SAUSA300_2160 | | SAUSA300_2639 | | SAUSA300_0672 | | SAUSA300_2077 | | SAUSA300_0217 | | SAUSA300_1717 | | SAUSA300_1220 | | SAUSA300_0350 | | SAUSA300_2156 | | | SAUSA300_2279 | | --- | | SAUSA300_1514 | | SAUSA300_2459 | | SAUSA300_2036 | | SAUSA300_0104 | | SAUSA300_2336 | | SAUSA300_0317 | | SAUSA300_2331 | | SAUSA300_0195 | | SAUSA300_1200 | | SAUSA300_0093 | | SAUSA300_1888 | | SAUSA300_2050 | | SAUSA300_1457 | | SAUSA300_1253 | | SAUSA300_0473 | | SAUSA300_0507 | | SAUSA300_1148 | | SAUSA300_2322 | | SAUSA300_0450 | | SAUSA300_1995 | | SAUSA300_1455 | | SAUSA300_0755 | | SAUSA300_1121 | | SAUSA300_0333 | | SAUSA300_0658 | | SAUSA300_2264 | | SAUSA300_2326 | | SAUSA300_0691 | | SAUSA300_2106 | | SAUSA300_2308 | | SAUSA300_2452 | | SAUSA300_2445 | | SAUSA300_0238 | | SAUSA300_0878 | | SAUSA300_1170 | | SAUSA300_2509 | | SAUSA300_2599 | | SAUSA300_2575 | | SAUSA300_1175 | | SAUSA300_0334 | | SAUSA300_2515 | | SAUSA300_2547 | | SAUSA300_2261 | | SAUSA300_2271 | | SAUSA300_2559 | | SAUSA300_2530 | | SAUSA300_1992 | | SAUSA300_2640 | | SAUSA300_2563 | | SAUSA300_0444 | | SAUSA300_1969 | | SAUSA300_0645 | | SAUSA300_2310 | | SAUSA300_0137 | | SAUSA300_1424 | | SAUSA300_1174 | | SAUSA300_0858 | | SAUSA300_0095 | | SAUSA300_0928 | | SAUSA300_0653 | | SAUSA300_2300 | | | SAUSA300_2490 | | --- | | SAUSA300_1797 | | SAUSA300_2566 | | SAUSA300_2216 | | SAUSA300_2303 | | SAUSA300_0577 | | SAUSA300_1722 | | SAUSA300_1708 | | SAUSA300_0519 | | SAUSA300_0605 | | SAUSA300_2248 | | SAUSA300_2245 | | SAUSA300_0654 | | SAUSA300_2218 | | SAUSA300_0954 | | SAUSA300_0803 | | SAUSA300_2247 | | SAUSA300_2571 | | SAUSA300_0373 | | SAUSA300_0066 | | SAUSA300_0114 | | SAUSA300_1204 | | SAUSA300_1434 | | SAUSA300_2438 | | SAUSA300_2437 | | SAUSA300_0804 | | SAUSA300_1946 | | SAUSA300_1968 | | SAUSA300_1433 | |

**Supplementary Table 2.** *S. aureus* strains analyzed in this work.

| **Strains** | **Genome size (Mbp)** | **Features^a^** | **References** |
| --- | --- | --- | --- |
| JH1 (USA100) | 2.93 | Vancomycin sensitive MRSA isolate | {Mwangi, 2007 #72} |
| JH9 (USA100) | 2.93 | VISA isolate evolved from JH1 | {Mwangi, 2007 #72} |
| FPR3757  (USA300) | 2.92 | Causes community-acquired infections in the USA, Canada, and Europe | {Diep, 2006 #27} |
| MRSA252 (USA200) | 2.9 | Epidemic MRSA | {Holden, 2004 #75} |
| Mu3 (USA100) | 2.9 | Representative hetero-VISA strain | {Hiramatsu, 1997 #73} |
| Newman (ST8 MSSA) | 2.88 | Isolated from a human infection | {Baba, 2008 #71} |
| Mu50 (USA100) | 2.82 | VISA, isolated in 1997 | {Kuroda, 2001 #8} |
| MSSA476 (ST1 MSSA) | 2.82 | Invasive community-acquired MSSA | {Holden, 2004 #75} |
| N315 (USA100) | 2.82 | MRSA, isolated in 1982 | {Kuroda, 2001 #8} |
| MW2 (USA400) | 2.82 | Community-acquired MRSA, caused fatal septicemia and septic arthritis, isolated in 1998. | {Baba, 2008 #71} |
| COL (Archaic) | 2.8 | Early MRSA isolate, resistant  to tetracycline and penicillin | {Gill, 2005 #76} |
| RF122 | 2.74 | Common strain associated with mastitis in cattle | {Herron, 2002 #70} |

^a^. MRSA, methicillin-resistant *S. aureus*; MSSA, methicillin-sensitive *S. aureus*; VISA, intermediate vancomycin sensitive *S. aureus*.
