# Supplementary figures and images for: Global analysis of transcriptional regulators in Staphylococcus aureus
Source: BMC Genomics. 2013 Feb 26;14:126. doi: 10.1186/1471-2164-14-126 (PMC3616918; doi:10.1186/1471-2164-14-126)

## Slide 1
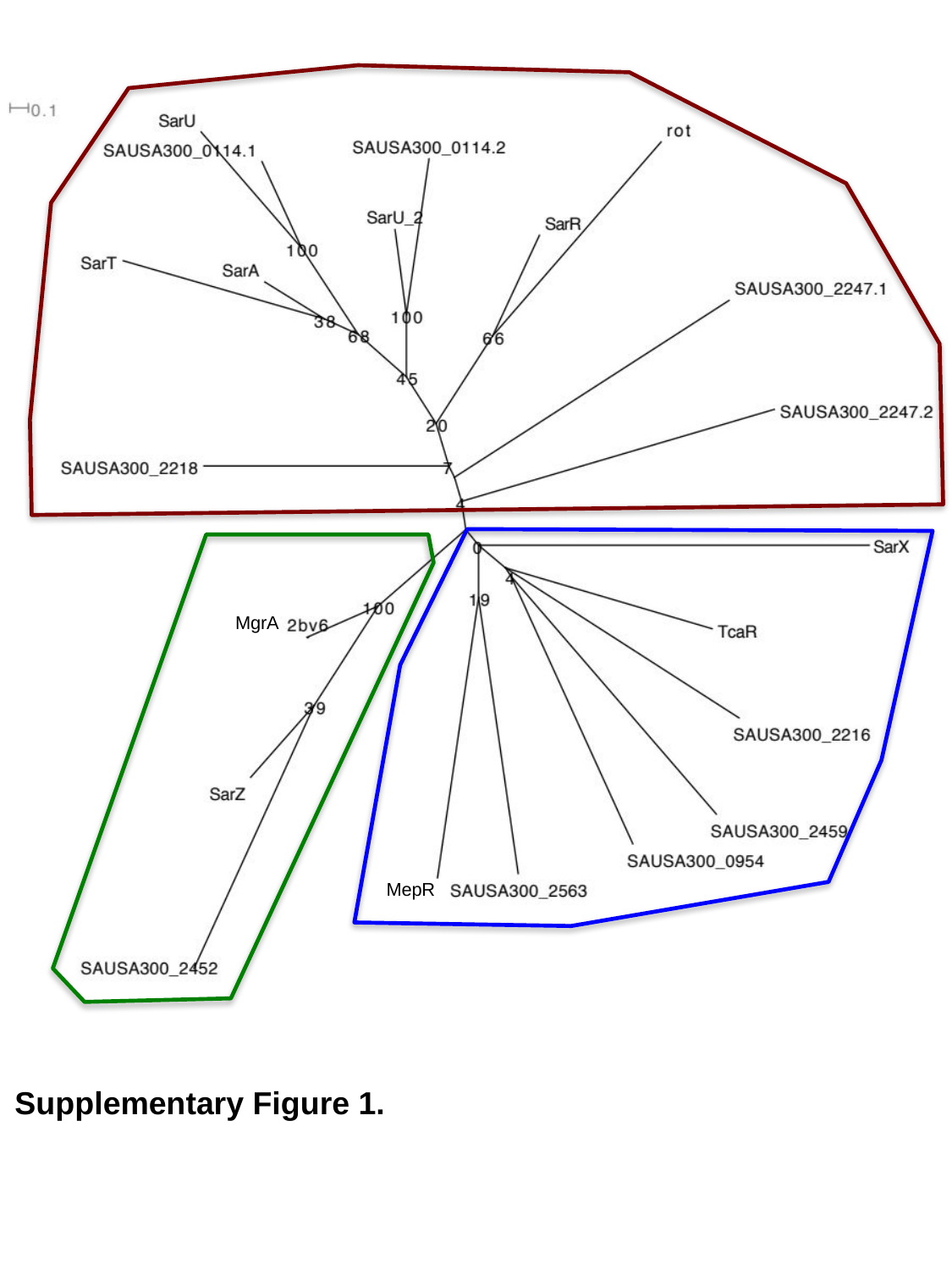

MgrA
MepR
Supplementary Figure 1.

Supplement: Additional file 2: Figure S1 — Phylogenetic tree for the MarR-like proteins. Sar and MarR proteins, along with uncharecterized MarR proteins, were aligned using the MgrA crystal (2BV6) structure as a template. Three clades are denoted, see text for details. SarU, SAUSA300_1114 and SAUSA300_2247 exhibit two MgrA-like domains and therefore are shown as duplicated in the tree. [file 1471-2164-14-126-S2.pptx]
